# Selective screening of patients with associated somatic diseases as a method of early detection of acromegaly

**DOI:** 10.14341/probl12699

**Published:** 2021-01-08

**Authors:** M. B. Antsiferov, V. S. Pronin, T. M. Alekseeva, O. A. Ionova, E. Y. Martynova, Yu. E. Poteshkin, N. A. Chubrova, K. Y. Zherebchikova

**Affiliations:** Endocrinological dispensary of Moscow Healthcare Department; Russian medical Academy of continuing professional education; Endocrinological dispensary of Moscow Healthcare Department; Endocrinological dispensary of Moscow Healthcare Department; LLC «Relevant medicine»; Pirogov Russian National Research Medical University (Pirogov Medical University); Moscow state medical University named after I.M. Sechenov (Sechenov University); Moscow state medical University named after I.M. Sechenov (Sechenov University)

**Keywords:** acromegaly, diagnostics, selective screening

## Abstract

**Backgraund:**

Backgraund: Acromegaly is a multi-organ disabling disease, the effectiveness of treatment of which directly depends on timely diagnosis. Latent course and delayed diagnosis increase the exposure of pathological hypersecretion of growth hormone and insulin-like growth factor-1, contributing to the development of irreversible systemic and metabolic changes in the body that negatively affect survival.

**Aims:**

Aims: The aim of the study was to clinically test a comprehensive diagnostic approach using selective screening to detect cases of acromegaly in patients with combined somatic diseases.

**Materials and methods:**

Materials and methods: The diagnostic search algorithm included a 2-stage questionnaire, expert assessment of the clinical status, laboratory and instrumental examination. The inpatient examination included the use of additional laboratory and instrumental methods and expert evaluation of the results obtained by filling out a doctor’s questionnaire. When the score was higher than 18 points, a more specific examination was performed: double determination of the insulin-like growth factor-1 level, oral glucose tolerance test with determination of the nadir of growth hormone value, and MRI of the brain with contrast enhancement. The diagnosis of acromegaly was made on the basis of personal data, expert assessment of the clinical status, results of laboratory and instrumental examinations.

**Results:**

Results: A survey of 1249 patients with combined systemic and metabolic disorders conducted using the point system allowed us to suspect acromegaly in 367 patients (29.4%), who were offered further examination. The majority of patients were previously seen by specialists for diabetes mellitus (79.3%) or thyroid pathology (10%). In the result of inpatient ­examination of 329 patients, 35 (10.6%) patients showed an increase in the blood level of IGF-I. In 19 patients, a persistent increase in the level of IGF-I was combined with the absence of GH suppression of less than 0.4 ng/ml against the background of glucose load. During MRI in 9 patients, pituitary adenoma was detected (in 2 — microadenoma and 7 — ­macroadenoma).

**Conclusions:**

Conclusions: As a result of the study, among the group of 1249 patients (mean age 58±13 years) with the presence of concomitant diseases, 9 newly identified patients with acromegaly were found who were prescribed adequate treatment. The introduction of selective screening technology into the practice of an endocrinologist will improve the effectiveness of diagnostic search for patients with acromegaly, more accurately assess the prevalence of the disease in Russia and the need for specialized medical care.

## Abstract (ru)

**Обоснование:**

Обоснование. Акромегалия является полиорганным инвалидизирующим заболеванием, эффективность лечения которого напрямую зависит от своевременной диагностики. Скрытое течение и запоздалый диагноз повышают экспозицию патологической гиперсекреции гормона роста и инсулиноподобного ростового фактора-1, способствуя развитию в организме необратимых системных и метаболических изменений, негативно влияющих на выживаемость.

**Цель:**

Цель. Клиническая оценка диагностического комплексного подхода с использованием селективного скрининга для выявления случаев акромегалии у пациентов с сочетанными соматическими заболеваниями.

**Методы:**

Методы. Алгоритм диагностического поиска включал двухэтапное анкетирование, экспертную оценку клинического статуса, проведение лабораторного и инструментального обследований. Стационарное обследование включало использование дополнительных лабораторно-инструментальных методов и экспертную оценку полученных результатов с заполнением анкеты врача. При наборе более 18 баллов проводили более специфическое обследование: двукратное определение уровня инсулиноподобного ростового фактора-1, оральный глюкозотолерантный тест с определением величины надира гормона роста, магнитно-резонансную томографию (МРТ) головного мозга с контрастным усилением. Диагноз «акромегалия» ставили на основании анкетных данных, экспертной оценки клинического статуса, результатов лабораторного и инструментального обследований.

**Результаты:**

Результаты. Проведенное по балльной системе анкетирование 1249 больных, имевших сочетанные системные и обменные нарушения, позволило заподозрить акромегалию у 367 пациентов (29,4%). Им было предложено дальнейшее обследование. Большинство из них ранее наблюдались у специалистов по поводу сахарного диабета (79,3%) или патологии щитовидной железы (10%). В результате стационарного обследования 329 пациентов у 35 (10,6%) больных было отмечено повышение в крови уровня инсулиноподобного ростового фактора-1. У 19 больных стойкое повышение его уровня сочеталось с отсутствием подавления гормона роста менее 0,4 нг/мл на фоне нагрузки глюкозой. При проведении МРТ у 9 больных была выявлена аденома гипофиза (у 2 — микроаденома и у 7 — макроаденома).

**Заключение:**

Заключение. В результате проведенного исследования 1249 пациентов (средний возраст 58±13 лет) с наличием сопутствующих заболеваний обнаружено 9 впервые выявленных больных акромегалией, которым было назначено адекватное лечение. Внедрение технологии селективного скрининга в практику работы эндокринолога позволит повысить эффективность диагностического поиска больных акромегалией, оценить распространенность заболевания в России и потребность в специализированной медицинской помощи.

## BACKGROUND

Acromegaly is a severe neuroendocrine disease that steadily leads to a decrease in the quality and duration of life of patients, which is a consequence of the cumulative negative effects of excessive concentrations of growth hormone (GH) on the human body. In the absence of appropriate treatment, the mortality rate of patients with acromegaly is 2-4 times higher than the average in the population. Independent predictors of untimely death include oncological diseases, cardiovascular disorders, arterial hypertension, diabetes mellitus, and an active stage long duration [1-5].

With the consideration of the multiplicity of clinical and pathomorphological options of the course of acromegaly, it should be noted that external somatic disease manifestations, as a rule, are not only delayed, but also not always pronounced. According to J. W. Hong et al., only 35% of patients have definitive changes in the facial skeleton, an increase in the size of soft tissues, hands, and feet [6]. More often, doctors have to deal with a mild form of acromegaly (micromegaly), which is observed in differentiated somatotropinomas consisting of densely particulated cells. The tumor is characterized in slow intracellar growth without signs of invasion and a tendency to recurrence. The form is characterized in late onset, moderate hormonal and proliferative activity, and minor orofacial and acral changes. In the retrospective study analysis L. B. Butz et al. have found that 47% of patients with acromegaly had a close to normal level of GH with a significant increase in the concentration of insulin-like growth factor-1 (IGF-1) and the presence of somatotropinoma [7]. The insidious nature of the disease progression and delayed diagnosis contribute to the development of multisystemic, metabolic and psychosomatic disorders in patients, which are manifested by a variety of clinical symptoms that complicate timely diagnosis. Incomplete clinical knowledge on acromegaly and the lack of a qualified diagnostic search system affect a significant increase in the period from the first manifestations of the disease to its detection (timelag). According to epidemiological data, 54% of patients timelag is more than 10 years, and 37% of patients for over 15 years, which significantly worsens the prognosis of the disease, which is inversely correlated with chronological age of patients, duration of the active stage of the disease and GH levels [8-11].

In addition to skeletal changes and mass effect, the most common clinical manifestations of acromegaly are arterial hypertension; cardiomyopathy, manifested by concentric myocardial hypertrophy with the development of diastolic dysfunction, valvular insufficiency and cardiac failure; respiratory failure due to obstructive sleep apnea, pulmonary emphysema and pneumosclerosis; nodular (diffuse-nodular) goiter; polyposis and intestinal tract diverticulosis. The leading issue in acromegaly is a violation of carbohydrate metabolism, the degree of incidence of which in the older age group reaches 50-60%. The prevalence of diabetes mellitus, nodular goiter, arterial hypertension, and cardiomyopathy correlates with the duration of the active stage of acromegaly [12-16]. According to F. Golkowski et al., already 3 years after the debut of acromegaly, one of the above complications is detected [17].

In addition, patients with acromegaly have 3.4 times greater risk of developing malignancies compared to the general population. The high incidence of colorectal cancer, breast and thyroid cancer has been proven. Oftimes, patients have multifocal tumors of various tissues. Thus, it was noted that the greatest number of extrapituitary neoplasias in acromegaly is observed against the background of symptomatic diabetes mellitus, the main pathogenetic links of which ( hyperinsulinemia and insulin resistance) increase cancer mortality rates [18-21].

Thus, the cumulative pathological effect of autonomous hyper-GР on the body leads to the development of multiple morphofunctional changes that contribute to early disability and untimely death of patients with acromegaly. The implication is that the disease early diagnosis and appropriate treatment, careful monitoring of possible complications play a crucial role in the optimal management of such patients and improving their survival [22-24].

According to international registries, the prevalence of acromegaly varies in different countries from 28 to 137 cases per 1 million population and depends on the age of debut, environmental conditions of residence, awareness of general practitioners and the quality of the prophylactic medical examination. In the Russian registry in 2020, there are 4114 patients with acromegaly, which corresponds to an average prevalence of 35.6 cases per 1 million population (Table. 1) [25–33].

**Table:** Table 1. Prevalence of acromegaly according to various national registers

Countries	Number of cases of ambulations (per 1 million population)
Belgium	21–125
Bulgaria	48
Brazil	50
Great Britain	86
Germany	70
Spain	16–76
Italy	26–210
Poland	79
Portugal	12,8
Russia	31,5
the USA	78–182
Uzbekistan	14
Finland	120
France	40–130

On average, the onset of the disease occurs in the IV-V decades of life, when, on the one hand, a pathogenic pool of natural and induced mutations accumulates in the cells, and on the other hand, involutional changes begin to prevail in the body, leading to the development of multisystemic dystrophic and neoplastic processes. An additional contribution to the aetiopathogenesis of acromegaly is made by poor environmental living conditions. So, according to the results of the investigation, S. Cannavo et al., the prevalence of acromegaly in environmentally pristine and industrially polluted areas of Italy was 26 and 210 patients per 1 million population, respectively, which confirms the mutagenic involvement of adverse environmental factors [34, 35].

The incidence and prevalence of acromegaly increase significantly with age. According to T. Burton et al., the incidence of acromegaly averages 11 new cases a year per 1 million population in the United States. Thus, in the age group under 17 years, this figure was 3-8 cases per 1 million, while in people over 65 years of age, the incidence increased to 9-18 cases per 1 million of the population. A similar trend persists when assessing the prevalence of this pathology: with an average of 78 cases per 1 million population, the prevalence of acromegaly in the groups of people under 17 years of age and over 65 years of age was 29-37 and 148-182 cases per 1 million population, respectively [36].

At the same time, it should be mentioned that: the registration of patients by the number of ambulations does not correspond to the real epidemiological situation in the region, which, in our opinion, is due to several reasons. Firstly, due to the lack of effective medical control, only a small part of patients go to medical institutions. Secondly, the awareness of doctors of other specialties regarding the clinical symptoms of acromegaly is not always at the adequate level. And thirdly, highly sensitive methods of diagnosing the disease are not everywhere applied. Significant progress in the detection of this pathology is associated with the introduction into clinical practice of methods for determining IGF-1, the increased content of which in the blood is a priority when establishing diagnosis [37].

It is noted in T.J. Reid et al. paper that over the past 25 years, the efficiency of detecting acromegaly has not undergone significant changes. In the majority of patients, the diagnosis is made at the at advanced stage of illness with a deliberately limited therapeutic maneuver and a negative prognosis [38]. The tardive diagnosis initially degrades the health condition of patients, since by the time of its detection the disease, as a rule, has already passed the “brink of no return” in its pathological development, sharply limiting the possibilities of its supervision. In this regard, when finding the case, doctors have to deal not only with its root cause, but also with multiple systemic and metabolic complications, the number of which inevitably increases as the latent period of the course enchances. We cannot bring up upon the pharmacoeconomic aspects, since the cost of treating complications is much higher than the cost of the prior disease follow-up [39].

All of the above indicates that the quality and life expectancy of patients with acromegaly depends to a large extent on the timely diagnosis. A paradoxical situation has arisen when the current techniques of treatment that provide patients with a full and fruitful life cannot be completely used due to the low efficiency of diagnostic services. For many unrecognized patients, the question of when to get access to appropriate treatment has become vital. The existing urgent problem of timely diagnosis of acromegaly initiated the development of programs for active mass or selective population screening, the specific characteristics and the first results of which are presented below.

Mass screening to identify patients with acromegaly includes the following programs

Selective screening is aimed at identifying acromegaly among patients with definitive associated diseases:

Regarding the preliminary results of the ongoing studies, it can be noted that any form of active updating of national registries deserves respect, since they contribute to the identification of an additional number of new patients and the improvement of diagnostic searching techniques. According to P.W. Rosario et al., even the primary introduction of symptomatic questionnaires into outpatient practice allowed for better diagnosis and an increase in the prevalence of acromegaly to 290 cases per million population [40].

Due to the developed computer programs for the analysis of the geometric arrangement of sign points on photographic images of the face, it has become possible to significantly increase the percentage of detected patients in comparison with the expert opinion, especially with a mild form of acromegaly. According to R.E. Miller et al., the diagnostic accuracy of mathematical processing of photographs is 86%, while medical diagnostics is only 26%. Similar investigations have shown that the diagnostic accuracy of computer models is 72% compared with expert diagnostics (63%) and conclusions of general practitioners (42%) [41–44]. The same applies to studying the possibilities of diagnosing the disease by assessing the acoustic characteristics of the voice in patients with acromegaly, which is characterized in a hoarse and low sound [45]. The previously conducted search for patients with acromegaly among patients with arterial hypertension or carpal tunnel syndrome did not bring the desired results due to the low specificity of signs.

Projects to identify acromegaly among patients with impaired carbohydrate metabolism have been successful. In the investigation by P.W. Rosario et al., among 2270 patients with type 2 diabetes mellitus aged 20 to 70 years, 3 patients with acromegaly were identified, which made it possible to increase the prevalence of the disease to 480 cases per 1 million population. In K. Suda et al. paper, the examination of 317 patients of the older age group (60.7 ± 14.2 years) with type 2 diabetes mellitus and impaired glucose tolerance revealed 2 cases of acromegaly. It is significant that as the age of the surveyed cohort increases, the percentage of detection of acromegaly also increases, amounting to 0.13 and 0.63%, respectively. These data confirm that acromegaly is more common among patients of the older age group with impaired carbohydrate metabolism [48, 49].

The results of the mass screening (DETECT study) conducted by H.J. Schneider et al. in 2008 are widely known. Total determination of the IGF-1 level in 6773 outpatients (age group 18+) made it possible to identify 7 patients with acromegaly, which increased the prevalence of the disease to 1034 cases per million population [46]. The disadvantage of this project can include the lack of a guiding vector and, as a result, large financial costs, limiting the possibility of its widespread implementation. Detection rate - 0.1.

The presented results of pilot epidemiological projects indicate that the actual prevalence of acromegaly is 15–20 times higher than officially registered, and, therefore, there is a large percentage of undiagnosed patients who do not receive specialized medical care. In this regard, early diagnosis of acromegaly remains an urgent public health problem, the solution of which will significantly increase the medical benefits efficacy.

For this purpose, for the first time in Russia in 2013–2015 the non-interventional cross-sectional study ACROSCREEN was carried out, in which the diagnostic search for patients was carried out among patients with the associated diseases, most often found in acromegaly (diabetes mellitus, nodular (diffuse-nodular) goiter, arterial hypertension, myocardial hypertrophy, sleep apnea syndrome, polyps and diverticula of the gastrointestinal intestinal tract, uterine myoma, endometrial polyps, polycystic ovary syndrome, prostatic hyperplasia, osteoarthropathy, spinal deformities, carpal tunnel syndrome, oncology diseases in the medical history).

## AIMS

The purpose of the study is to clinically test a diagnostic procedure for detecting cases of acromegaly in patients with associated somatic diseases.

## MATERIALS AND METHODS

## Study design

The study was observational. No interventions in routine clinical practice were performed within the study. Validation criteria For inclusion in the study, patients over the age of 18 years with somatic diseases associated with acromegaly were considered. By the exclusion criterion the patients with a previously confirmed diagnosis of acromegaly were meant.

## Realization conditions

The search for study participants was carried out among 1249 patients who came for an appointment and were observed in connection with various diseases at the State Budgetary Healthcare Institution of the Endocrinological Dispensary of Moscow City Health Department and FSAEI HE of I.M. Sechenov First Moscow State Medical University of the Ministry of Health of the Russian Federation (Sechenov University). The patients who, according to the results of the questionnaire, scored 18 or more points and were included into the risk group, were offered further participation in the study.

## Study duration

The duration of the study inclusion period was from December 2013 to September 2015.

## Description of the medical intervention

The study was conducted in 2 stages. At the first stage, a questionnaire survey of patients who came to the hospital and were observed there in connection with various diseases was carried out (Table 2). Then the patients selected for the study underwent the in-patient examination, which included additional laboratory and instrumental techniques and the expert assessment of the results obtained with filling out a doctor’s questionnaire (Table 3). At the 2nd stage, the more specific examination was carried out: double determination of IGF-1, oral glucose tolerance test to determine the value of nadir GH, MRI of the brain with contrast enhancement. On the basis of personal data, expert assessment of the clinical status, the results of laboratory and instrumental examinations, the patients were diagnosed with «acromegaly».

**Table:** Table 2. Questionnaire for the patient with suspected acromegaly

Have you noticed any changes in your appearance over the last year:	No	Yes	I don’t know
Fingers clubbing, increasing the size of rings, gloves	0	4	
Increasing shoe size	0	4	
Nose enlargement	0	3	
Enlargement of lips	0	3	
Enlargement of superciliary arch and orbital bone	0	3	
Increasing the size of the jaws with occlusal disharmony and interdental space expansion	0	3	
Increasing head circumference (headgear size)	0	3	
Do you suffer from:
Headache	0	2	
Hyperhidrosis	0	2	
Pain, stiffness, and decreased flexibility in the joints	0	2	
Increase in arterial pressure	0	2	
Increased blood sugar	0	2	
Labored breathing and performance impairment	0	2	
Fingertips numbness	0	2	
Visual deterioration	0	2	
Snoring or shortness of breath strokes while sleeping	0	2	
Hair overgrowth, the appearance of skin formations (birthmarks, nevi, condylomas, fibroids, neurofibromas, lipomas, hemangiomas)	0	2	
Presence of skin folds on the face and hairy part of the head	0	2	
Breast discharges	0	2	
Increased attention of others to your appearance	0	2	
Have you previously and/or currently got:
Thyroid disorders	0	1	
Gynecological diseases (hysteromyoma, endometrial polyps)	0	1	
Breast disease	0	1	
Intestinal tract polyp(s)	0	1	
Tumor diseases of other organs	0	1	
Gynecological and obstetric history:
Menstrual irregularities and a pregnancy pathology in the history	0	1	
Multiple fetation or closely spaced pregnancy	0	1

 

**Table:** Table 3. Doctor’s Questionnaire for Detecting Patients with Acromegaly

The presence of the sign symptoms	None	Yes	ND
Characteristic change in appearance and increase in the size of the limbs	0	1	
Increased tongue size and dysphrasia	0	1	
Skin thickening	0	1	
Hyperhidrosis	0	1	
Presence of benign skin lesions	0	1	
Arterial hypertension	0	1	
Myocardial hypertrophy (rhythm disturbance, conduction abnormality)	0	1	
Nodular (diffuse-nodular) goiter	0	1	
Sleep apnea syndrome	0	1	
Osteoarthropathy, spinal deformity	0	1	
Carpal tunnel syndrome	0	1	
Polyps and GIT diverticula	0	1	
Carbohydrate metabolism disorder (IGT, diabetes mellitus)	0	1	
Uterine myoma, endometrial polyps, polycystic ovary syndrome	0	1	
Chronic cystic masticatory	0	1	
Laboratory examination data (if carried out earlier)
Hyperglycemia	0	1	
Hypercholesterolemia	0	1	
Hypertriglyceridemia	0	1	
Hypercalcemia	0	1	
Instrumental examination data (if carried out earlier)
Cordis ultrasound investigation: myocardial hypertrophy, reduced cardiac output, diastolic, systolic dysfunction	0	1	
Ultrasound of the thyroid gland: increase in volume, the presence of palpable abnormality	0	1	
Medical ultrasound of the abdominal organs: increase in the size of internal organs, the presence of cysts, sclerotic tissue changes	0	1	
Total	

The laboratory and instrumental examination comprised the following:

The content of IGF-1 in blood serum was determined by the chemiluminescence technique on an automatic analyzer Liaison (DiaSorin, Italy) or by the immunoradiometric technique using the IGF-1 reagent kits produced by Immunotech (France), depending on the clinical facility. The GH level in the blood was determined using a solid phase chemiluminescence enzyme immunoassay on an automatic analyzer IMMULITE 2000 (Diagnostic Products Corporation, the USA).

Contrast-enhanced brain MRI was performed on the high-field tomograph (> 2 T) (in the presence of biochemically confirmed acromegaly).

## The outcomes registration methods

Confirmation of the diagnosis of «acromegaly» was:

## Ethical expert review

The study was conducted in accordance with the ethical principles embodied in the Declaration of Helsinki [51]. The Diagnostic Search Protocol was approved by the ethics committees of both research centers and met all the regulatory requirements. The screened population included all patients who were familiar with the objectives and techniques of the study (ID number on the website of ClinicalTrials.gov: NCT02020967) and have signed informed consentе. Since the study was of a non-interventional nature, no safety assessment was provided.

## Statistical analysis

Sampling size calculation principles: the study did not include a formal sampling size.

Techniques for the Data Statistical Methods. In the investigated cohort, analyses were performed to assess the primary and secondary endpoints. The data from the questionnaires of patients and researchers were analyzed for the establishment or exclusion of the «acromegaly» diagnosis and compared with each other using the F-test statistic to determine statistically significant differences. Quantitative characteristics were assessed by the mean and standard deviation. Differences were considered statistically significant at a significance level of p <0.05 To determine the most pathognomonic subjective and sign symptoms of acromegaly, the logistic regression model and the discriminant analysis model were investigated. A stepwise algorithm (PROC STEPDISC, SAS / STAT® Software) was used for discriminant analysis; roc statements were used to build a logistic regression model; roccontrast - for PROC LOGISTIC SAS procedure. The final model was assessed using a receiver operating characteristic curve (ROC) to determine the specificity of the model in order to distinguish between patients with acromegaly and patients who have no acromegaly.

## RESULTS

## Subjects of the investigation (participants)

As a result of the questionnaire survey of 1249 patients, further participation in the study was offered to 367 (29.4%) patients who scored 18 points or more, of which 38 patients (10.4%) refused further participation. 329 patients (89.6%) have passed to the examination stage. The average age of the examined cohort was 58 ± 13 (46–72) years. The number of men - 54 (16%), women - 275 (84%).

As a result of the initial processing of individual registration cards, it was revealed that the majority of patients were observed for a long time by various specialists for type 2 diabetes mellitus (79.3%) or thyroid pathology (10%). Among other diseases, cardiopathy (18.5%), osteoarthropathy (8.5%), neoplasms (22.8%), gynecological diseases (1.8%) were more common (Table 4)

**Table:** Table 4. Sex-age-specific characteristics of patients

Characteristics	With acromegaly(n=9)	Without acromegaly(n=320)	Total (n=329)
Age (years old)	58,1	58,0	58,0
Average (SD)	8,22	13,11	12,99
Median	58	61	60
Range	46–72	22–90	22–90
Women, n (%)	7 (77,8)	268 (83,8)	275 (83,6)
Men, n (%)	2 (22,2)	52 (16,3)	54 (16,4)

 

## The study primary results

An increase in the IGF-1 level above the age-appropriate normal value was observed in 35 patients (10.6%). The presence of «biochemical acromegaly», confirmed by a persistent increase in the IGF-1 level and the value of the GR nadir> 0.4 ng/ml, was detected in 19 cases. Of these - 17 patients underwent the contrast enhanced brain MRI, as a result of which a pituitary adenoma was detected in 9 patients (2 - microadenoma and 7 - macroadenoma). In the group of patients with identified GH-secreting pituitary adenoma, the mean IGF-1 value was 580 ± 336 ng/ml (in the group without acromegaly - 156 ± 62 ng/ml, p <0.05).

In 8 patients with «biochemical acromegaly», no hormonally active adenoma was found intra or extrapituitary, which indicates the need for further case follow-up.

So, as a result of a diagnostic search among patients with an average age of 58 ± 13 years and with the presence of concomitant diseases, 9 patients (2 men and 7 women) (age 58 ± 13 years) were newly diagnosed with «acromegaly». Previously, 5 of the newly diagnosed patients with acromegaly were treated for diabetes mellitus, 2 - for thyroid pathology, 1 - for osteoarthropathy and 1 - for cardiopathy. The duration of the disease prior the establishing diagnosis of acromegaly was 6.6 (1–16) years. The late diagnosis of the disease was indicated by the presence of somatic and metabolic complications, as well as the presence of a pituitary macroadenoma in the vast majority of patients. After detection of acromegaly, all patients were prescribed pathogenetic treatment (curative adenomectomy or primary medicamentous therapy with somatostatin analogs and dopamine agonists).

According to the results of the questionnaire, statistically significant signs that distinguish patients with acromegaly were: typical changes in appearance and an increase in the size of the limbs (p = 0.025), osteoarthropathy (p = 0.0163), hysteromyoma, endometrial polyps (p = 0.049). In the logistic regression model, the only statistically significant indicator of acromegaly was an increase in facial soft tissues (“enlargement of lips”). The inequality ratio for this indicators was 7.34, with a 95% confidence interval (CI) of 1.38–39.11 (p = 0.0196). In the discriminant analysis model, 3 statistically significant indicators were identified that increase the risk of the diagnosis of «acromegaly»: «typical changes in appearance and an increase in the size of the limbs», «enlargement of the lips», «headache». The developed model of discriminant analysis suggested acromegaly in 5 out of 9 patients with a confirmed diagnosis. The area of the ROC curve, which measures the overall ability of the model to discriminate between patients with acromegaly and those without it, was 0.8294. This means that the sensitivity of the model to predicting acromegaly in this study is 55.56%, specificity is 90% (taking into account the prediction of the absence of acromegaly in 288/320 patients without acromegaly) (Fig. 1).

**Figure:**
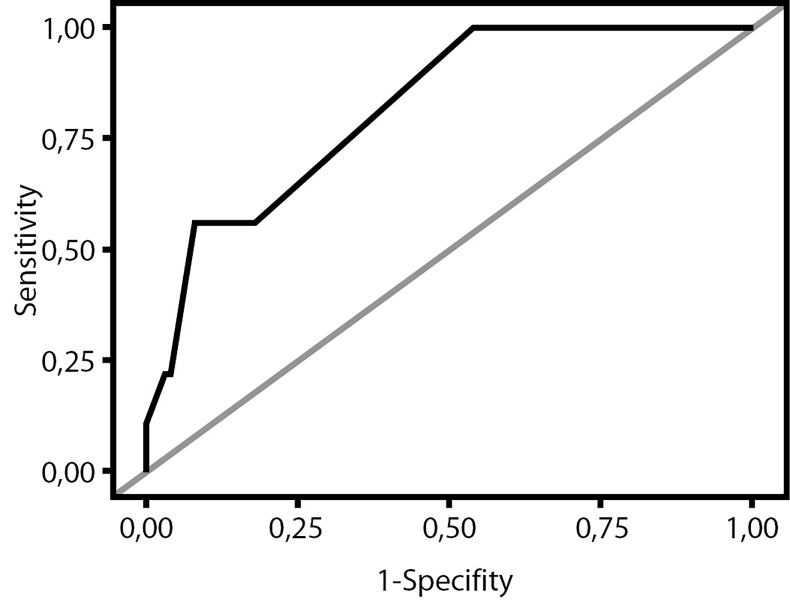
Fig. 1. Receiver operating characteristic curve based on the discriminant analysis model.

## DISCUSSION

## The Summary for the Study Primary Results

The data obtained confirm that the real prevalence of acromegaly, especially among the older age group with associated diseases, is significantly higher than that in the analysis of ambulations, which emphasizes the urgency of the problem of timely diagnosis of the pathology [2, 10]. The implementation of the ACROSCREEN program is aimed at identifying a risk group that includes patients with a specific set of clinical, somatic and anamnestic signs and requiring additional diagnostic search. Pursuant to the international experience and our data, the risk group for acromegaly should include patients over 50 years old with impaired carbohydrate metabolism, nodular (diffuse-nodular) goiter, as well as arterial hypertension, cardiopathy, sleep apnea, arthropathy, benign or malignant neoplasias. It should be noted that it is problematic to objectively judge the general epidemiological situation in the Russian Federation on the basis of only one study, therefore, to identify comparable data, similar projects are required in various regions of the country.

## Discussion of the Study Primary Results

Based on the results of the work performed, thanks to targeted questioning of patients from the risk group using questionnaires, expert assessment, laboratory and instrumental experimental techniques, the authors were able to identify a fairly high percentage of newly diagnosed patients with acromegaly - 0.72% (9 out of 1249). In similar studies of P.W. Rosario et al. and K. Suda et al. this figure was 0.13 and 0.63%, respectively. High detectability, in our opinion, is due to the selective study of the age group at risk with combined diseases associated with acromegaly, the indispensable inclusion of an endocrinologist in the questionnaire process and an expert assessment of the results obtained. In the future, it is planned to improve the approch of active diagnostic search, especially since this direction is actively developing in world practice. So, in 2016, the results of the population study ACROSCORE were presented, which revealed a strong association of objective signs, indicating the maximum likelihood of acromegaly in the presence of type 2 diabetes mellitus, hyperhidrosis, diffuse (diffuse-nodular) goiter, colorectal polyps, diastema, carpal tunnel syndrome [52].

## STUDY LIMITATIONS

Given the multiplicity of incoming diagnoses, it is difficult to identically assess the prevalence of acromegaly in the Russian Federation. To calculate the approximate prevalence of acromegaly among patients with associated diseases, the authors identified a cohort of patients with diabetes mellitus. Among this nosology (ICD), the prevalence of acromegaly was 1.9% (5 patients out of 261). Further calculations were carried out according to the scheme proposed by P.W. Rosario et al. [47]. If bearing in mind that, according to the national register, at the beginning of 2016 there were about 4 million adult patients with type 2 diabetes in Russia, then 1.9% of the total number is about 76 thousand patients with acromegaly. Since carbohydrate metabolism disorder in acromegaly occurs in about 55% of cases, the total number of patients is approximately 138 thousand. This figure is 33.5 times higher than the number of patients with acromegaly included in the Russian register in 2020 [33], and indicates that the overwhelming majority of potential patients do not receive specialized medical care.

It should also be considered that in each of these studies, a certain number of patients with «biochemical acromegaly» and an undetected pituitary tumor were found. In the cohort of P.W. Rosario, there were 3 of such patients [47], in the group of K. Suda - 18 people [49], in the sample of H.J. Schneider - 4 [46], in our study - 8 people. Further examination of these patients category will make it possible to establish whether we are talking about an ectopic tumor or the disease early stage.

## CONCLUSION

Late diagnosis of acromegaly, especially with a mild form of the disease (micromegaly), contributes to the development of multiple somatic and metabolic disorders that reduce the quality and duration of life. The use of the ACROSCREEN two-stage selective screening program with preliminary identification of the risk group made it possible to identify 9 (0.72%) patients with acromegaly among patients who had previously received long-term inadequate treatment for another pathology. The real prevalence of acromegaly in the Russian Federation among residents of the older age group with associated diseases significantly exceeds the known rates of ambulations, which requires widespread introduction of targeted diagnostic search technology into the clinical practice.
